# Differential transcriptome analysis of the disease tolerant Madagascar–Malaysia crossbred black tiger shrimp, *Penaeus monodon* hepatopancreas in response to acute hepatopancreatic necrosis disease (AHPND) infection: inference on immune gene response and interaction

**DOI:** 10.1186/s13099-019-0319-4

**Published:** 2019-07-26

**Authors:** Tze Chiew Christie Soo, Sridevi Devadas, Mohamed Shariff Mohamed Din, Subha Bhassu

**Affiliations:** 1 Department of Genetics and Molecular Biology, Institute of Biological Sciences, Faculty of Science, University of Malaya, 50603 Kuala Lumpur, Malaysia; 2 Centre for Research in Biotechnology for Agriculture (CEBAR), Research Management and Innovation Complex, University of Malaya, 50603 Kuala Lumpur, Malaysia; 3 Selangor Fisheries Biosecurity Centre, Department of Fisheries, Malaysia, KLIA, 63000 Sepang, Selangor Malaysia; 4 Institute of Bioscience, Universiti Putra Malaysia, 43400 Serdang, Selangor Malaysia; 5 Department of Veterinary Clinical Studies, Faculty of Veterinary Medicine, Universiti Putra Malaysia, 43400 Serdang, Selangor Malaysia

**Keywords:** *Penaeus monodon*, Acute hepatopancreatic necrosis disease (AHPND), Transcriptome analysis, Shrimp innate immune response, PAMPs, DAMPs

## Abstract

**Background:**

*Penaeus monodon* is the second most widely cultured marine shrimp species in the global shrimp aquaculture industry. However, the growth of *P. monodon* production has been constantly impaired by disease outbreaks. Recently, there is a lethal bacterial infection, known as acute hepatopancreatic necrosis disease (AHPND) caused by *Vibrio parahaemolyticus* AHPND strain (*Vp*_AHPND_), which led to mass mortalities in *P. monodon*. Unfortunately, there is still insufficient knowledge about the underlying immune response of *P. monodon* upon AHPND infection. The present study aims to provide an insight into the antibacterial immune response elicited by *P. monodon* hepatopancreas towards AHPND infection.

**Methods:**

We have employed high-throughput RNA-Seq technology to uncover the transcriptome changes of *P. monodon* hepatopancreas when challenged with *Vp*_AHPND_. The shrimps were challenged with *Vp*_AHPND_ through immersion method with dissected hepatopancreas samples for the control group (APm-CTL) and treatment group at 3 (APm-T3), 6 (APm-T6), and 24 (APm-T24) hours post-AHPND infection sent for RNA-Seq. The transcriptome de novo assembly and Unigene expression determination were conducted using Trinity, Tgicl, Bowtie2, and RSEM software. The differentially expressed transcripts were functionally annotated mainly through COG, GO, and KEGG databases.

**Results:**

The sequencing reads generated were filtered to obtain 312.77 Mb clean reads and assembled into 48662 Unigenes. Based on the DEGs pattern identified, it is inferred that the PAMPs carried by *Vp*_AHPND_ or associated toxins are capable of activating PRRs, which leads to subsequent pathway activation, transcriptional modification, and antibacterial responses (Phagocytosis, AMPs, proPO system). DAMPs are released in response to cell stress or damage to further activate the sequential immune responses. The comprehensive interactions between *Vp*_AHPND_, chitin, GbpA, mucin, chitinase, and chitin deacetylase were postulated to be involved in bacterial colonization or antibacterial response.

**Conclusions:**

The outcomes of this research correlate the different stages of *P. monodon* immune response to different time points of AHPND infection. This finding supports the development of biomarkers for the detection of early stages of *Vp*_AHPND_ colonization in *P. monodon* through host immune expression changes. The potential genes to be utilized as biomarkers include but not limited to C-type lectin, HMGB1, IMD, ALF, serine proteinase, and DSCAM.

**Electronic supplementary material:**

The online version of this article (10.1186/s13099-019-0319-4) contains supplementary material, which is available to authorized users.

## Background

The shrimp aquaculture industry contributes as a vital economic pillar in many countries worldwide especially those with middle- or low-level economies. The industrial production is majorly dominated by countries in the Asian region, for example, China, India, Malaysia, Thailand, Vietnam, Indonesia, and Ecuador [1]. The three main commercial shrimp or prawn species in the industry are *Penaeus monodon*, *Litopenaeus vannamei*, and *Macrobrachium rosenbergii*.

The shrimp species involved in the present study is *P. monodon*. As described by [2, 3], *P. monodon* was officially and taxonomically classified by Fabricius in the year 1798. *P. monodon* is commonly called black tiger shrimp due to its morphological appearance of dark brown colour with blackish hue when placed in the pond.

Although there have been great technology and yield improvements in the global shrimp aquaculture industry, shrimp diseases have remained a heavy hurdle to the future sustainability of the industry [4]. The shrimp diseases, which are mostly viral, bacterial, and fungal diseases are able to cause great economic losses to the shrimp farmers when disease outbreaks occur. This makes the shrimp aquaculture a high-risk investment industry [1].

A newly emerged shrimp bacterial disease, known as acute hepatopancreatic necrosis disease (AHPND) has caused massive mortalities and great economic losses in shrimp aquaculture industries of many Asian and South American countries. *P. monodon* and *L. vannamei* are susceptible to AHPND infection [5]. AHPND is caused by a new strain of *Vibrio parahaemolyticus* marine Gram-negative bacteria, known as *Vp*_AHPND_, which is capable of infecting and inhabiting shrimp gut cavity and hepatopancreas [5]. According to the research findings of [6], *V. parahaemolyticus* is observed to possess the ability to colonize the digestive tract and stomach of *P. monodon* through interaction with chitin molecules. After successful colonization, toxins and enzymes are then released by the bacteria to infect and damage the shrimp.

*Vp*_AHPND_ carries one or more extrachromosomal plasmids (pVA1) about 70kbp that encode *Photorhabdus* insect-related (Pir) binary toxins homologues called PirA_vp_ and PirB_vp_. After propagation in the shrimp gut cavity, the *Vp*_AHPND_ bacteria then release deadly toxins, PirA_vp_ and PirB_vp_, which damage the hepatopancreas of the shrimp resulting in shrimp deaths [5, 7]. AHPND infection can usually cause shrimp mortalities from 40 to 100% within 10–35 days. Some gross signs of AHPND infection include slow growth, empty stomach and midgut, pale white hepatopancreas, and lethargy [5, 8]. Other *Vibrio* species have also been observed to be carrying pVA-like plasmids, which demonstrates the transmitability of toxic plasmids found in *Vp*_AHPND_ [9]. This issue further increases the concern of AHPND infection as a serious threat to the shrimp aquaculture industry. Shrimps have been experimentally infected with *Vp*_AHPND_ through various methods, such as immersion, per os (feeding), reverse gavage, and cohabitation [10].

In recent years, the study of omics through bioinformatics technologies and techniques is an increasing trend among scientists. Along with next-generation sequencing technological advances and bioinformatics developments, RNA sequencing (RNA-Seq)-based transcriptome analysis has become a better and more affordable option for transcriptomic studies [11]. RNA-Seq transcriptome analysis is a newer and more efficient method for comparative differential gene expression study.

The present study mainly focuses on analysing the underlying physiological immune response of *P. monodon* hepatopancreas upon AHPND infection. This involves identifying the different types of vital immune response towards AHPND infection. More specifically, the insight into the correct chronological order of the immune response events triggered can be obtained. This provides deeper knowledge in the study of host–pathogen interactive relationship and development of accurate diagnosis method for detection of different stages of *Vp*_AHPND_ infection.

## Methods

### Pre-challenge preparations

*Vibrio parahaemolyticus*, AHPND strain (*Vp*_AHPND_) bacteria were isolated from the dissected organs of 10 moribund *P. monodon* suspected with AHPND outbreak. The AHPND-infected samples were validated through observation of gross clinical signs (empty midgut, empty stomach, atrophied and pale hepatopancreas) and AP3 polymerase chain reaction (PCR) detection method [12]. The digestive systems of dissected AHPND-infected shrimps were placed aseptically into tubes containing 10 ml of tryptic soy broth (TSB+) (Merck, Darmstadt, Germany) supplemented with 2% sodium chloride (NaCl). The tubes were incubated at 28 °C for 18 h at 120 rpm for the enrichment of bacteria. The enriched broth cultures were streaked on thiosulfate citrate bile salt (TCBS) agar to select the green-colony forming *V. parahaemolyticus*. The isolation of green colonies was conducted on tryptic soy agar (TSA+) (Merck, Darmstadt, Germany) supplemented with 2% NaCl. The enrichment was done through incubation at 28 °C for 18 h at 120 rpm. The bacterial cultures were stored at − 80 °C using cryovial (CRYOBANK™) and revived for downstream applications. The validated positive strains were referred to as *Vp*_AHPND_ strains KS17.S5-1, KS17.S5-2, and KS17.S9-2 [13].

### Experimental challenge of *P. monodon* with *Vp*_AHPND_

One of the previously obtained *Vp*_AHPND_ strain KS17.S5-1 [13] was revived and prepared for the experimental challenge. Juvenile commercial pond cultured disease tolerant crossbred shrimps between 13th generation Madagascar *P. monodon* strain and 5th generation local *P. monodon* strain with body length from 15 to 20 cm were acclimatized for a week in designed experimental setup. The shrimps in the treatment group were then infected with *Vp*_AHPND_ (2 × 10^6^ cfu/ml) through a modified immersion method similar to descriptions by [14]. Sterile TSB+ broth was added instead of *Vp*_AHPND_ for the shrimps in the control group. The experimental challenge was conducted with three replicates for both treatment and control groups, and 27 shrimps placed in each tank. The important organs (hepatopancreas, muscle, stomach, gut, and haemolymph) were dissected from one shrimp collected from each tank for every time interval of 0, 3, 6, 12, 24, 36, and 48 h post-AHPND infection and stored at − 80 °C.

### RNA extraction and sequencing

Total RNA was extracted from the hepatopancreas samples of infected shrimps at 3 (APm-T3), 6 (APm-T6), and 24 (APm-T24) hours post-AHPND infection together with non-infected control shrimp (APm-CTL) using TransZol Up Plus RNA Kit (Transgen Biotech, Beijing, China). The extracted RNA samples were then treated with DNase and sent for cDNA library preparation and sequencing using BGI-SEQ 500 Sequencer.

### Quality assessment and transcriptome de novo assembly

The raw sequencing reads were filtered based on several criteria, which include removal of reads with adaptors, removal of reads with more than 5% of unknown bases, and removal of low-quality reads (percentage of bases which quality is lesser than 15 is more than 20% in a read). The filtered reads quality metrics were assessed based on Q30, the rate of bases which quality is greater than 30. The filtered clean reads were stored in FASTQ format. The clean reads were then used for de novo assembly through Trinity software [15] with PCR duplication removed.

### Determination of assembled Unigene expression level and functional annotation

For the Unigene expression determination, clean reads were mapped to Unigenes using Bowtie2 software [16] followed by gene expression level determination using expectation maximization (RSEM) software [17], and principle component analysis (PCA) using princomp (function of R software) [18]. The differentially expressed genes (DEGs) were identified based on PossionDis [significant DEGs: fold change ≥ 2.00, false discovery rate (FDR) ≤ 0.001]. The distribution of assembled Unigene expression level and number of DEGs at different post-infection time intervals was compared.

The assembled Unigenes were then functionally annotated through alignment of the Unigenes to nucleotide (NT), non-redundant protein sequence (NR), Gene Ontology (GO), Cluster of Orthologous Groups of proteins (COG), SwissProt, Kyoto Encyclopedia of Genes and Genomes (KEGG), and InterPro functional databases. Basic Local Alignment Search Tool (BLAST) software [19] was used for Unigene alignment with KEGG database; BLAST2GO software [20] was used with NR annotation to obtain GO annotation; InterProScan5 software [21] was used to obtain InterPro annotation. Species distribution of the assembled Unigenes was determined based on NR annotation. For the DEGs, the KEGG analysis was functionally enriched using phyper, a function of R software [18].

### Identification of immune-related differentially expressed Unigenes

From the COG, GO, and KEGG analyses obtained, the DEGs involved in immune-related biological functions and pathways were identified and listed down. An immune-related keyword search was also conducted among the Unigenes with high fragments per kilobase million (FPKM) values and DEGs. The pattern of the immune DEGs’ activation or repression upon AHPND infection at different time points was identified and possible interactions between them were deduced.

### Expression profile validation through qRT-PCR

The RNA-Seq expression profiles obtained for selected immune-related DEGs (C-type lectin, IMD, ALF, and HMGB1) were validated through the Real Time Reverse Transcription Polymerase Chain Reaction (qRT-PCR) technique with three biological replicates and three technical replicates each across post-AHPND infection time points. The qRT-PCR primers were designed using PrimerQuest Tool software (https://sg.idtdna.com/PrimerQuest/Home/Index). First strand cDNA synthesis was conducted using TransScript^®^ One-Step gDNA Removal and cDNA Synthesis SuperMix (TransGen Biotech, Beijing, China). The qRT-PCR experiments were conducted using Agilent Technologies Stratagene Mx3005P instrument and GoTaq^®^ qPCR Master Mix kit (Promega, Madison, Wisconsin, USA). The qRT-PCR reaction consisted of 10 µl GoTaq^®^ qPCR 2X Mix, 500 nM forward primer, 500 nM reverse primer, and 2 µl template cDNA. The qRT-PCR cycling program used was 95 °C for 2 mins, 40 cycles at 95 °C for 15 s, and 55 °C for 1 min. Elongation factor 1-alpha (EF1a) gene was selected to be the internal control reference gene. The primer sequences designed were listed in (Additional file 1: Table S4). The Ct values obtained were then analysed using Livak’s 2^ddCt^ relative quantification method [22]. The results were statistically validated through One-Way Analysis of Variance (One-Way ANOVA) analysis and subsequent post hoc Duncan test using SPSS software Version 22.

## Results

### Transcriptome sequencing, quality assessment, and assembly

The transcriptome sequencing using BGI-SEQ 500 sequencing platform for AHPND-infected (APm-T3, APm-T6, and APm-T24) and non-infected control (APm-CTL) *P. monodon* hepatopancreas extracted RNA samples was successfully conducted. The RNA Sequencing (RNA-Seq) generated a total of 320.57 Mb raw reads, 312.77 Mb clean reads, and 31.27 Gb clean bases. The quality assessment of the clean reads at the level of Q30 was overall satisfactory and deemed as high quality with quality percentages greater than 90% (Q30) (Additional file 1: Table S1). After transcriptome assembly, a total of 48662 Unigenes was obtained from the data of APm-CTL, APm-T3, APm-T6, and APm-T24 with a total length of 56,085,297 bp, an average length of 1152 bp, an N50 of 2289 bp, and a GC content of 44.10% (Additional file 1: Table S2).

### Comparison of Unigene expression levels and DEGs

**Fig. 1:**
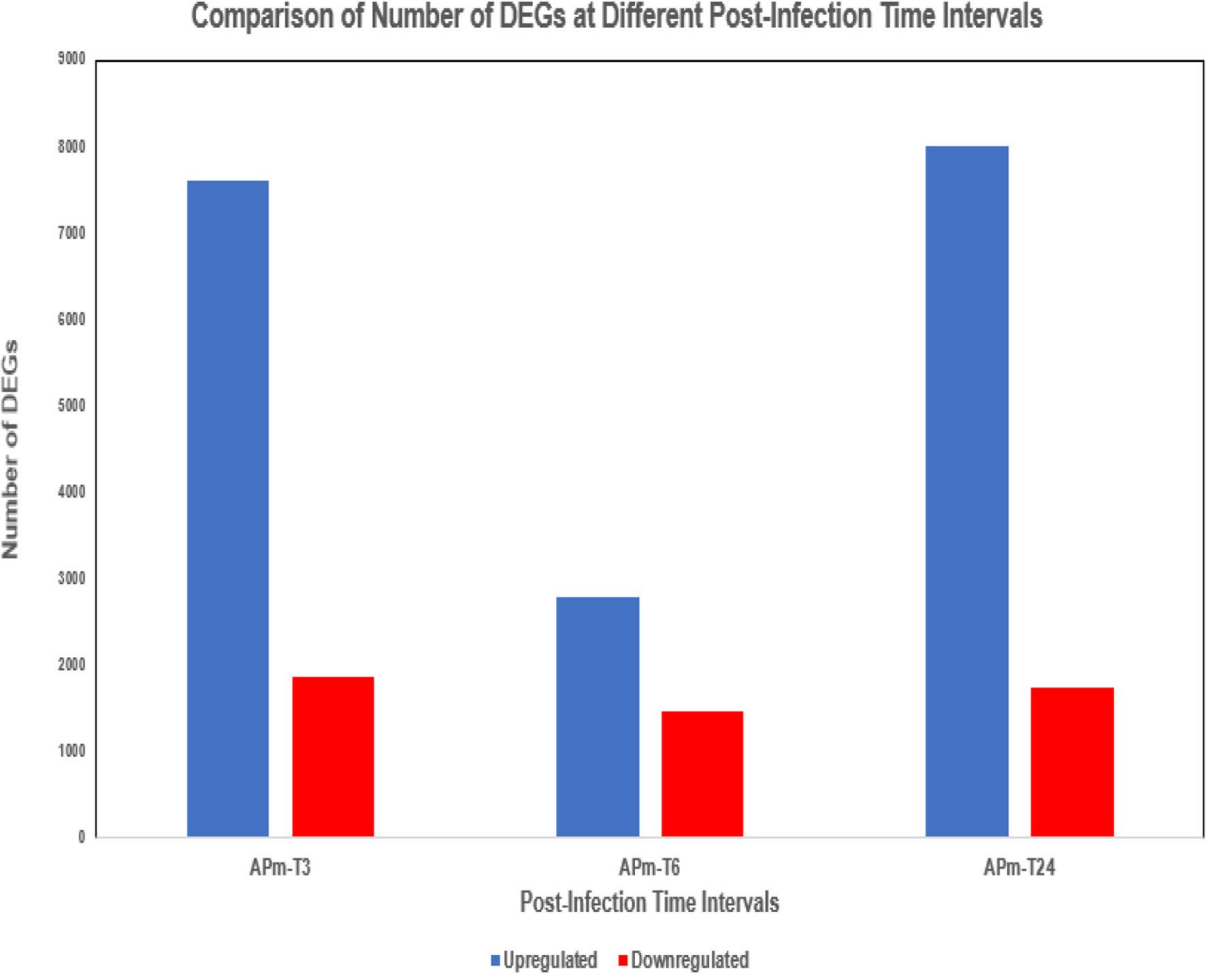
Comparison of number of DEGs at different post-infection time intervals

Based on the distribution of Unigenes across FPKM values in Additional file 1: Figure S1, most of the Unigenes were identified to possess fragments per kilobase million (FPKM) values ranging from 0.01 to 100. The transcripts per million (TPM) values were also calculated to be majorly in the range of 0.01 to 200. From the FPKM and TPM distribution across length of Unigenes graph plotted (Additional file 1: Figure S2), the majority of the gene expression was focused in Unigenes with length from 301 to 5000 bp. A brief comparison of the number of differentially expressed genes (DEGs) between the post-AHPND infection time intervals was also conducted. APm-T3 had 7605 upregulated DEGs and 1869 downregulated DEGs; APm-T6 had 2802 upregulated DEGs and 1464 downregulated DEGs; APm-T24 had 7998 upregulated DEGs and 1752 downregulated DEGs. It was clearly observed that APm-T6 had a significantly lower amount of upregulated DEGs when compared to APm-T3 and APm-T24 (Fig. 1).

### Functional annotation of Unigenes and DEGs

The Unigenes were functionally annotated using selected seven functional databases and showed results of non-redundant protein sequence (NR) (24,709; 50.78%), nucleotide (NT) (18,622; 38.27%), SwissProt (21,388; 43.95%), Cluster of Orthologous Groups of proteins (COG) (11,636; 23.91%), Kyoto Encyclopedia of Genes and Genomes (KEGG) (20,696; 42.53%), Gene Ontology (GO) (4510; 9.27%), and InterPro (16,438; 33.78%) (Additional file 1: Table S3). The species distribution of the Unigenes using NR annotation results was shown in Additional file 1: Figure S3. The *P. monodon* Unigenes were successfully matched with *Daphnia pulex* (8.39%), *Tribolium castaneum* (4.12%), *Pediculus humanus corporis* (2.89%), and *Branchiostoma floridae* (2.39%). This indicated the close phylogenetic relationship of *P. monodon* to these species. The remaining 82.21% of Unigenes were matched to other species mostly due to limited genetic information in crustaceans.

The 11636 Unigenes assigned to COG annotation were classified into 25 functional protein families (Additional file 1: Figure S4). The Unigenes were mainly involved in “carbohydrate transport and metabolism” (1741 Unigenes), “cell wall/membrane/envelope biogenesis” (1224 Unigenes), and “signal transduction mechanism” (1098 Unigenes).

From the GO analysis obtained (Additional file 1: Figure S5), 4510 Unigenes were assigned to 60 different functions under categories of biological process, cellular component, and molecular function. More specifically, there were 563 Unigenes involved in “response to stimulus”, 370 Unigenes in “signalling”, and 49 Unigenes in “receptor activity”.

Besides that, from the KEGG analysis obtained (Additional file 1: Figure S6), 42 top matched pathways were displayed. Some of the important pathways detected were “signal transduction” (3878 Unigenes), “infectious diseases: bacterial” (2186 Unigenes), and “immune system” (1672 Unigenes).

Furthermore, in the KEGG pathway enrichment analysis based on DEGs (Additional file 1: Figure S7), several notable changes occurred following the different time intervals of post-AHPND infection. These included the detection of “cytosolic sensing pathway”, “sulfur relay system”, and “apoptosis” during APm-T3. At APm-T6, “cell adhesion molecules”, “phagosomes”, “ascorbate and aldarate metabolism”, “Rap 1 signalling pathway”, “adipocytokine signalling pathway”, and “platelet activation” were detected. In addition, APm-T24 was observed to involve “DNA replication and repair” (homologous recombination, non-homologous end joining, nucleotide/base excision repair, mismatch repair), “linoleic acid biosynthesis”, “arachidonic acid metabolism”, “apoptosis”, and “cytosolic DNA sensing pathway”.

Based on the gene functions of the Unigenes identified in the GO analysis conducted (Additional file 1: Figure S5), Unigenes were preliminarily screened out according to their functioning in relation to pathogenic or immune response such as adhesion, signalling, and binding. Similarly, this preliminary screening was conducted on KEGG analysis (Additional file 1: Figure S6) and DEGs-based functional enriched KEGG analysis (Additional file 1: Figure S7). The screened Unigenes were confirmed with comparison to other functional annotations. Finally, the immune-related DEGs that were expressed in all treatments were selected from the preliminarily screened Unigenes and further discussed.

### Identification and selection of immune-related DEGs

**Fig. 2:**
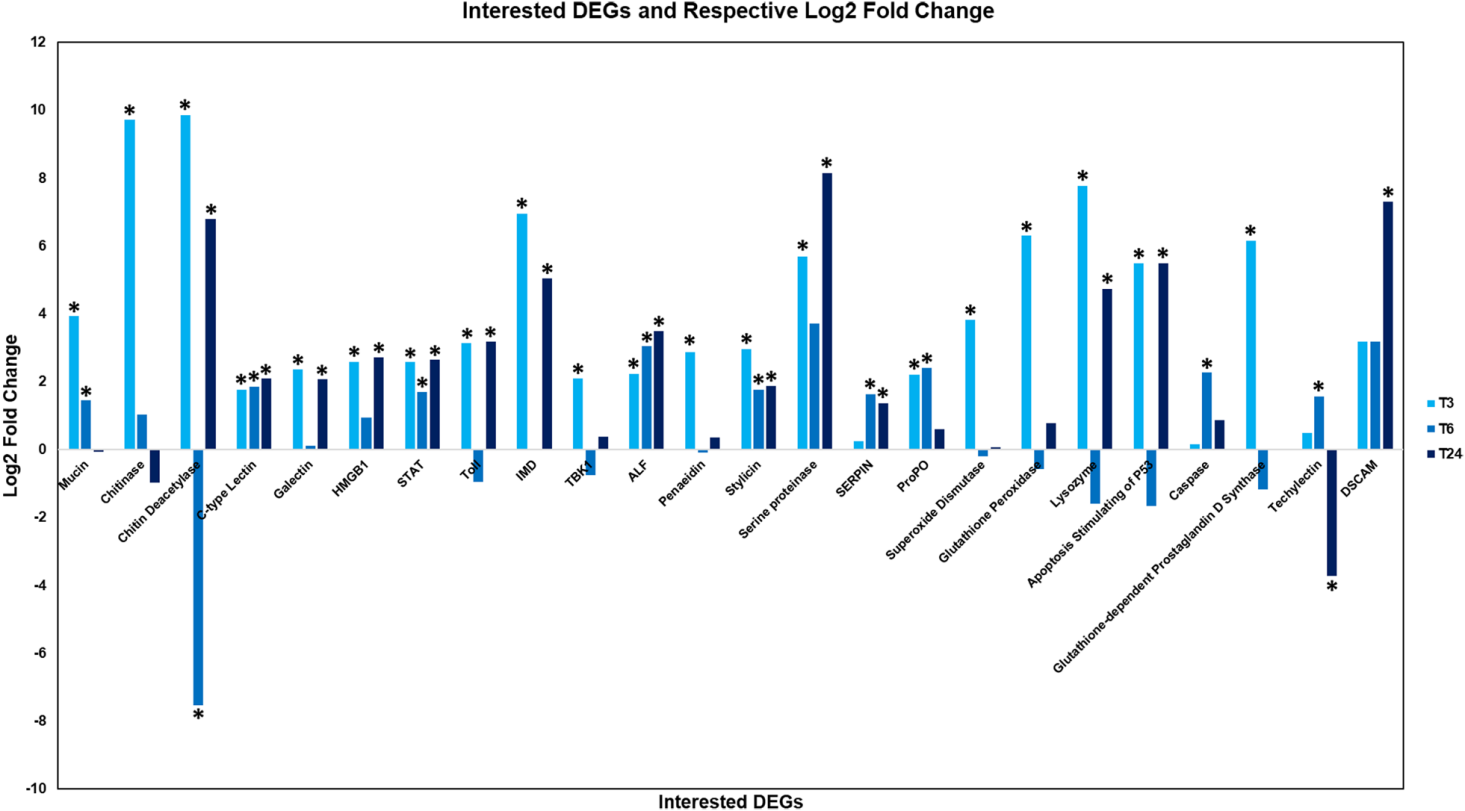
Interested DEGs related to immune response of *P. monodon* with their respective log2 fold change. The statistically significant log2 fold changes were labelled with “*”. Legends were T3: APm-T3, T6: APm-T6 and T24: APm-T24

**Fig. 3:**
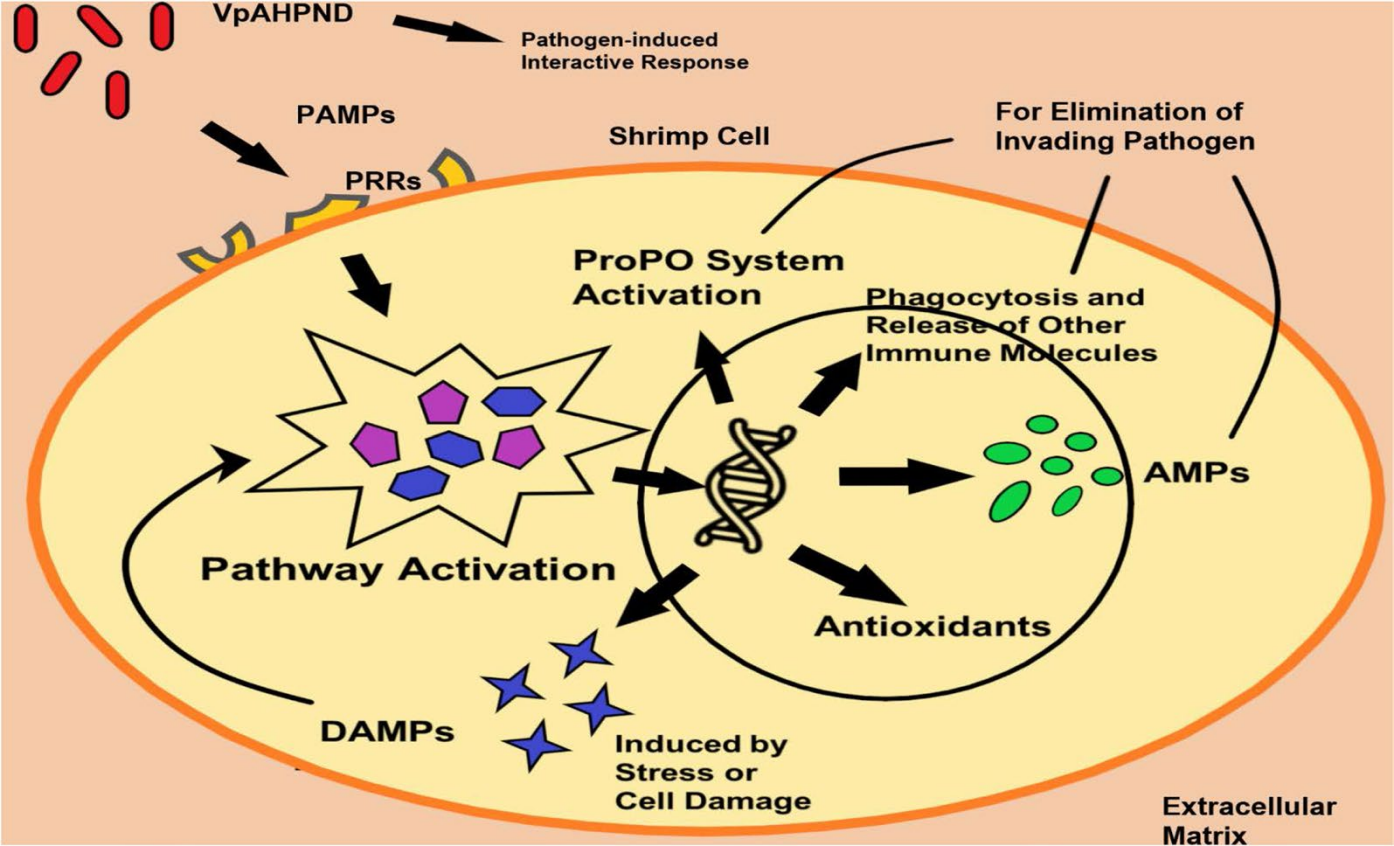
The inferred immunological response pattern of *P. monodon* hepatopancreas in response to AHPND infection

All of the important screened and selected immune-related DEGs were listed down in Table 1 with respective category, gene id, annotated homologous identity, primary annotation, secondary annotation, and log2 fold changes. The 24 selected DEGs were involved in the innate immune response of *P. monodon* towards AHPND infection. These selected DEGs have important functions in pathogen-induced interactive response (mucin, chitinase, and chitin deacetylase), pattern recognition receptors (PRRs) and damage-associated molecular patterns (DAMPs) (C-type lectin, galectin, and high mobility group box 1 (HMGB1)), immune or signalling pathway activation [signal transducer and activator of transcription (STAT), Toll, immune deficiency (IMD), and tank-binding kinase 1 (TBK1)], antimicrobial peptides (AMPs) [anti-lipopolysaccharide factor (ALF), penaeidin, and stylicin], prophenoloxidase (proPO) system [serine proteinase, serine proteinase inhibitor (SERPIN), and proPO], antioxidation (superoxide dismutase, glutathione peroxidase), phagocytosis (lysozyme, apoptosis stimulating of p53, and caspase), and other immune-related responses [glutathione-dependent prostaglandin d synthase, techylectin, and down syndrome cell adhesion molecule (DSCAM)]. The selected DEGs and their respective log2 fold changes across the three post-AHPND infection time points were shown in Fig. 2. The overall immunological response pattern of *P. monodon* hepatopancreas during AHPND infection in chronological order was then inferred based on the selected DEGs as shown in Fig. 3.

**Table 1:** Interested immune-related DEGs with their annotated identities, primary annotation, secondary annotation and log2 fold changes

Category/gene ID	Annotated homologous identity	Primary annotation	Log2 fold change		
			T3	T6	T24
Pathogen-induced interactive response genes					
Unigene 10589	Mucin	KEGG: K10955 intestinal mucin-2	3.940*	1.457*	− 0.0589
CL1511.Contig2	Chitinase	KEGG: K01183 chitinase	9.723*	1.018	− 0.963
Unigene 6305	Chitin deacetylase	NR: ABW74152.1| chitin deacetylase 9 [*Tribolium castaneum*]	9.862*	− 7.539*	6.792*
PAMPs and DAMPs genes					
Unigene 5450	C-type Lectin	NR: AAZ29608.1|/1.17917e-129/C-type lectin [*Penaeus monodon*]	1.766*	1.841*	2.097*
Unigene 274	Galectin	KEGG: GB18324; galectin 2	2.354*	0.126	2.072*
Unigene 17462	HMGB1	NR: ADQ43366.1|/3.82992e-161/HMGBa [*Litopenaeus vannamei*]	2.570*	0.926	2.719*
Pathway-related genes					
Unigene 21346	STAT	KEGG: K11224 signal transducer and activator of transcription 5B	2.569*	1.693*	2.655*
Unigene 1234	Toll	NR: ABO38434.1|/0/Toll receptor [*Penaeus monodon*]	3.135*	− 0.939	3.170*
Unigene 18449	IMD	NR: ACL37048.1|/2.11556e-113/IMD [*Litopenaeus vannamei*]	6.949*	0	5.044*
Unigene 17197	TBK1	KEGG: K12652 TANK-binding kinase 1-binding protein	2.079*	− 0.748	0.376
AMPs genes					
Unigene 16331	ALF	NT: EF523560.1/2e-78/*Penaeus monodon* anti-lipopolysaccharide factor isoform 1 (ALFPm1) gene, complete cds	2.215*	3.051*	3.478*
Unigene 19432	Penaeidin	NR: ACH70378.1 penaeidin 3 [*Penaeus monodon*] & ACQ66008.1 penaeidin 5 antimicrobial peptide [*Penaeus monodon*]	2.867*	− 0.0884	0.353
Unigene 10242	Stylicin	NR: ABW24769.1 stylicine 2 [*Litopenaeus stylirostris*]	2.960*	1.749*	1.871*
proPO system genes					
Unigene 3984	Serine proteinase	NT: AY372186.1/0.0/Penaeus monodon serine proteinase mRNA, partial cds	5.672*	3.700	8.155*
CL3265.Contig1	SERPIN	NR: AHC06147.1|/0/serpin 3 [Penaeus monodon]	0.242	1.630*	1.352*
Unigene 3184	proPO	NR: AGI42860.1 prophenoloxidase 3 [Fenneropenaeus chinensis]	2.208*	2.395*	0.602
Antioxidation genes					
Unigene 3737	Superoxide dismutase	KEGG: K04565 superoxide dismutase, Cu–Zn family [EC:1.15.1.1]	3.826*	− 0.198	0.0722
Unigene 6270	Glutathione peroxidase	KEGG: K00432 glutathione peroxidase [EC:1.11.1.9]	6.298*	− 0.567	0.787
Phagocytosis-related genes					
Unigene 2116	Lysozyme	NR: ACZ63472.1i-type lysozyme-like protein 2 [*Penaeus monodon*]	7.762*	− 1.585	4.737*
CL1336.Contig2	Apoptosis stimulating of P53	KEGG: K17554 apoptosis-stimulating of p53 protein 1	5.478*	− 1.663	5.482*
Unigene 5149	Caspase	KEGG: K02186 caspase 2 [EC:3.4.22.55]	0.162	2.268*	0.874
Other immune-related genes					
Unigene 24702	Glutathione-dependent prostaglandin d Synthase	NR: AFJ11393.1|/3.09192e-104/glutathione-dependent prostaglandin d synthase [*Penaeus monodon*]	6.152*	− 1.170	0
Unigene 12001	Techylectin	SwissProt: sp|Q9U8W7|TL5B_TACTR/3e-65/Techylectin-5B OS = *Tachypleus tridentatus* PE = 1 SV = 1	0.497	1.565*	− 3.730*
Unigene 33847	DSCAM	KEGG: K06768 Down syndrome cell adhesion molecule-like protein 1	3.169	3.170	7.313*

The log2 fold change values that are labelled “*” are statistically significant based on PossionDis (FC ≥ 1, P value ≤ 0.05, FDR ≤ 0.001)

### Differential gene expression validation of immune-related DEGs through qRT-PCR

**Fig. 4:**
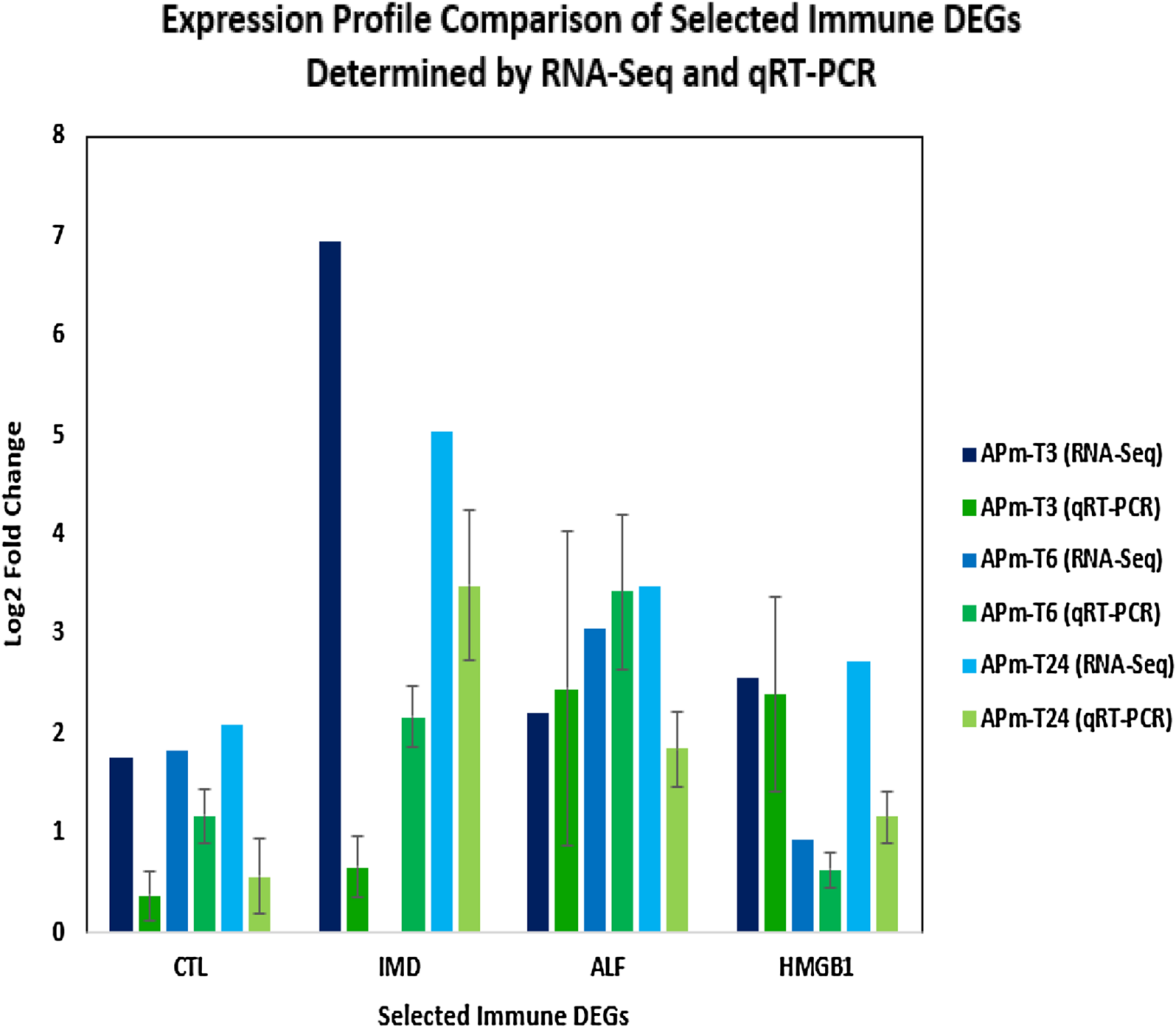
Expression profile comparison of selected immune DEGs determined by RNA-Seq and qRT-PCR. Selected immune DEGs abbreviations are as follows: *CTL* C-type lectin, *IMD* immune deficiency, *ALF* anti-lipopolysaccharide factor, *HMGB1* high mobility group box 1. Error bar indicates standard deviations among three biological replicates

The differential gene expressions of the selected immune-related DEGs identified in RNA-Seq were successfully validated using the qRT-PCR technique. Four Unigenes (C-type lectin, IMD, ALF, and HMGB1) were selected for the qRT-PCR analysis. The qRT-PCR results demonstrated similar trends to the RNA-Seq data in Fig. 4. The qRT-PCR results of individual Unigenes were shown in Additional file 1: Figures S8–S11. The results were proven to be statistically significant through One-Way Analysis of Variance (One-Way ANOVA) analysis and subsequent post hoc Duncan test (p < 0.05) (Additional file 1: Tables S5–S8). Even though the results from RNA-Seq and qRT-PCR analyses did not match perfectly, most probably because of sequencing bias, the gene expression patterns of immune-related DEGs across post-AHPND infection time points were generally validated by the qRT-PCR analysis.

## Discussions

### Transcriptome sequencing quality and general gene expression pattern

Based on Additional file 1: Figure S1, the majority of Unigenes detected was identified to possess fragments per kilobase million (FPKM) values ranging from 0.01 to 100 in all four treatments, APm-CTL, APm-T3, APm-T6, and APm-T24. This indicated that the Unigenes commonly displayed a low-level to mid-level gene expression. The Unigenes with FPKM values of > 100 were relatively rare. Whereas in Additional file 1: Figure S2, the FPKM and transcripts per million (TPM) values obtained were shown to be efficient normalization methods for the determination of Unigene expression levels with minimal read length bias.

The number of downregulated differentially expressed genes (DEGs) was similar between APm-T3, APm-T6, and APm-T24. However, it is interesting to observe that the number of upregulated DEGs was significantly lower in APm-T6 as compared to the other two treatments (Fig. 1). This can be due to the gene expression repression, DNA damage or cell damage that resulted from AHPND infection either from the direct influence of *Vp*_AHPND_ bacteria or the effect of released bacterial toxins. The invading bacteria are capable of influencing and reprograming host gene expression during host–pathogen interactions to achieve either beneficial or unfavourable effects towards bacterial propagation and persistence [23].

### Postulated interactive relationship between *Vp*_AHPND_, Chitin, GbpA, mucin, chitinase, and chitin deacetylase

The upregulated mucin, chitinase, and chitin deacetylase gene expressions in *P. monodon* hepatopancreas in response to AHPND infection (Fig. 2) suggests an interactive relationship with *Vp*_AHPND_ involving chitin. The ability of *Vibrio* species bacteria to interact with chitin molecules to achieve tolerance and adaptation is explained by the previously validated *Vibrio cholerae*-chitin interaction. Such interaction is important for purposes such as habitat selection and bacterial pathogenicity. The bacteria are capable of utilizing pili (for example, type IV pili) for chitin binding to colonize on the host surface, usually exoskeletons or intestinal cavities [24]. This takes into account the basic composition of chitin molecules, which is β-1,4-linked *N*-acetylglucosamine (GlcNAc) residues and the chemotaxis properties of *Vibrio* spp. [24]. GlcNAc-binding protein A (GbpA) is a vital protein released by *Vibrio* bacteria for binding with chitin subunits, GlcNAc [25]. GbpA’s interaction with chitin and associated components was also investigated in *M. rosenbergii* previously [26, 27].

The process of chitin binding triggers the activation of bacterial competence state or related pathogenicity through DNA acquisition and transformation [24]. *Vibrio*-chitin interactions at the cellular level can lead to the subsequent formation of biofilm on the attachment surface, which is a multicellular complex [24, 28]. The mucus layer is significantly affected by the gene expression of mucin, which is an organic constituent of mucus. Interestingly, intestinal mucin functions as a receptor for GbpA and induces its expression. Alternatively, the binding of GbpA to host cells also increases the production of mucus. Thus, there exists a cooperative upregulation mechanism between mucin and GbpA gene expressions [25, 29]. A similar condition is exhibited in *Pseudomonas aeruginosa*-mucin interactions [30].

There is also a possibility of host gene expression and translation mechanism hijacking by invading pathogens. A good example is shown by [25], by which Type III Secretion Systems (TTSS) was employed by *V. parahaemolyticus* bacteria for the delivery of effector proteins into host epithelial cells. Such effectors increase the cytotoxicity, enterotoxicity, and intercellular adherence of *V. parahaemolyticus* bacteria through alteration of host signalling proteins and regulation of cellular behaviour.

Chitinase is proposed and preliminarily validated to be functional in shrimp innate immune defence and phagocytosis, as exemplified by the newly analysed *L. vannamei* chitinase 5 (LvChi5) [31]. The biological immune defence role played by chitin deacetylase through chitin in shrimp is shown from its gene expression upregulation in a challenge experiment of *Exopalaemon carinicauda* against *V. parahaemolyticus* [32]. On the other hand, the expression of chitinase and chitin deacetylase enzymes are also observed in *Vibrio* spp. for the successful colonization, modification or degradation of chitins on the host epithelial cells [33].

Therefore, it can be postulated that the invading *Vp*_AHPND_ bacteria released GbpA protein, chitinase enzyme, and chitin deacetylase enzyme to interact with and colonize the chitin molecules found on surfaces of gut cavities or hepatopancreas in *P. monodon* shrimps. As a result, the *P. monodon* hepatopancreas, which is an important digestive gland, secreted chitinase and chitin deacetylase as a measure of early antibacterial response. There was also a cooperative upregulation of *Vibrio* GbpA protein and *P. monodon* mucin receptor gene expressions as part of the pathogen-induced interactive response and probable bacterial hijacking mechanism. All these were reflected by the upregulated expressions of mucin, chitinase, and chitin deacetylase in *P. monodon* hepatopancreas during AHPND infection.

### PAMPs, DAMPs, pathway activations, and AMPs

Pathogen-associated molecular patterns (PAMPs) and damage-associated molecular patterns (DAMPs) are important signals for the activation of innate immunity [34, 35]. PAMPs are associated with invading microorganisms and recognized by pattern recognition receptors (PRRs) found on host cells. This leads to the activation of signalling pathways and elevated expression of antimicrobial peptides (AMPs). On the other hand, DAMPs are released during stressed or damaged cell condition regardless of the presence or absence of pathogenic infection. PAMPs and DAMPs are known as “Signal 0 s” as they are always responsible as the initial molecules which bind to receptors triggering cascade reactions. They are mainly involved in host immune defence and apoptosis [34].

According to [34], PAMPs are usually microbial nucleic acids and membrane components. Examples of PAMPs are lipopolysaccharide (LPS), β-1,3-glucan (βG), and peptidoglycan (PG). PAMPs lead to the release of endogenous molecules (EMs) which contain DAMPs and other immune-related molecules [36]. PRRs can be C-type lectin receptors, Toll-like receptors (TLRs), and AIM2-like receptors (ALRs). Examples of DAMPs are high mobility group box 1 (HMGB1), galectin, uric acid, heparin sulphate, heat shock proteins, ATP, and S100 proteins. HMGB1 is one of the highly characterized DAMPs and largely expressed in the nucleus [34–36]. All these molecules contribute to the activation of shrimp innate immunity during pathogenic infection. Following this would be cell damage, haemocyte degranulation and necrosis, associated elevated level of phenoloxidase (PO) activity, and respiratory burst (RB) [36].

In the present study, during the different time points of post-AHPND infection, several genes functioning as PRRs (C-type lectin, galectin) and DAMPs (galectin, HMGB1) were identified to be upregulated (Fig. 2). PRRs were upregulated mainly during early times of infection whereas DAMPs were upregulated during later times of infection. The upregulation of these genes suggests that either the direct infection of *Vp*_AHPND_ bacteria at *P. monodon* hepatopancreatic cells or indirect pathogenic infection through toxin damage carried the bacterial signals known as PAMPs, which triggered the activation of PRRs and subsequent release of DAMPs.

The currently known shrimp immune signalling pathways vital for disease combating include Janus kinase-Signal transducer and activator of transcription (JAK-STAT) pathway, Immune deficiency (IMD) pathway, TLRs pathway, RNA interference (RNAi) pathway, c-Jun N-terminal kinase (JNK) pathway, and P38 mitogen-activated protein kinase (MAPK) pathway [4]. Among these pathways, TLRs, IMD, and JAK-STAT are the main pathways functioning in shrimp innate immune defence against microbial infection. The components of these pathways or their invertebrate homologues have been previously identified in different shrimp species proving the functionalities of the pathways in shrimps [37]. TLRs pathway mainly involves Toll, Spätzle, Pelle, MyD88, Cactus, and Dorsal. IMD pathway involves IMD, IκB kinase (IKK), and Relish. JAK-STAT pathway involves JAK and STAT [4].

A novel immune signalling pathway, known as cytosolic sensing pathway, which basically involves the detection of microbial cytosolic DNA (CDNs), activation of stimulator of interferon genes (STING) molecules, and subsequent expression of interferons and cytokines by tank-binding kinase 1 (TBK1)/STING complex. The mammalian STING has been proven to participate in host innate immune response [38]. For invertebrates, there was a recent publication by [39], which demonstrated the important function of *L. vannamei* STING (LvSTING) in shrimp antimicrobial innate immune defence through *V. parahaemolyticus* bacterial challenge.

The detection of upregulated Toll, IMD, STAT, and TBK1 gene expressions in *P. monodon* hepatopancreas during AHPND infection in the present study (Fig. 2) indicates the activation of corresponding TLRs, IMD, JAK-STAT, and cytosolic sensing pathways. The activation of these immune-related signalling pathways was validated by the similarly upregulated AMPs, which include anti-lipopolysaccharide factor (ALF), penaeidin, and stylicin (Fig. 2). This is because shrimp immune signalling pathways generally end with the production of AMPs to target, kill, and clean up invading pathogens. Examples of such AMPs are penaeidin (anti-bacterial and anti-fungal), ALF (anti Gram-negative bacteria), stylicin (antimicrobial), and crustin (anti-bacterial or anti-viral) [4].

These upregulated gene expressions that are related to PRRs, DAMPs, immune pathways, and AMPs suggest the occurrence of a continuous cascade of reactions initiated upon contact of bacterial PAMPs with shrimp cells. The successful activation of PRRs led to the activation and upregulation of genes in the order of immune pathway genes, AMP genes, and DAMP genes. Expressed or released DAMPs then functioned similarly to PAMPs to upregulate gene expressions of immune pathway genes and AMP genes until the elimination of invading pathogen or cancellation of cell stress and damage condition. This flow of reactions was described similarly as well in some previously published papers [4, 40, 41].

### ProPO system activation and phagocytosis

In crustaceans, the prophenoloxidase (proPO) system is an important mechanism of innate immune defence that involves a cascade of serine proteinases converting inactive proPO to active phenoloxidase (PO), thus causing downstream immune actions, such as toll pathway activation, immune gene synthesis, and melanisation [42]. Serine proteinase cascade activation can be triggered by microbial or fungal cell wall components, including LPS in Gram-negative bacteria, PG in Gram-positive bacteria, and βG in fungi. AMPs are released as a result of proPO system activation for pathogen elimination [42]. Melanisation response or cellular melanotic encapsulation is an effective immune response against invading pathogens, especially parasites. However, strong regulation of proPO system is needed as excessive activation will damage host cells. This is mostly done by regulatory proteins called SERPINs [42, 43]. Interestingly, there is evidence of interaction between proPO system and lysozyme, and possible inhibition of proPO’s conversion to PO by lysozyme through protein interaction [43].

In the present study, the gene expression upregulations of serine proteinase, proPO, and SERPIN were detected during AHPND infection of *P. monodon* (Fig. 2). The serine proteinase and proPO genes were upregulated initially at APm-T3 which led to the deduction that binding of *Vp*_AHPND_ bacterial LPS to serine proteinase cascades caused the conversion of proPO to PO and downstream reactions. The proPO gene expression was upregulated as well to support and sustain the cascade reactions of proPO system. This is supported by the upregulation of SERPIN at later time points of APm-T6 and APm-T24 as SERPIN functions as a negative regulator of the proPO system.

### Inferred immune response pattern of *P. monodon* in response to AHPND infection

The immune response of *P. monodon* hepatopancreas that was elicited upon AHPND infection is deduced to be a chronological event by which the immune-related receptors or proteins or genes were activated or upregulated systematically as shown in Figs. 2 and 3. The inference is made mainly based on the gene expression fold change pattern of involved immune-related genes across different post-AHPND infection time points. The concept of the inference is based on the general cellular innate immunity mechanism as described in some previously published papers [4, 40, 41].

During APm-T3, the initial interaction between *Vp*_AHPND_ bacteria and *P. monodon* hepatopancreatic cells resulted in the occurrence of pathogen-induced interactive response involving the upregulation of mucin, chitinase, and chitin deacetylase genes of *P. monodon*. At the same time, the PAMPs carried by *Vp*_AHPND_ bacteria activated the shrimp cell membrane PRRs which subsequently led to the activation of immune pathways. The cascade reactions ended with transcriptional activation or repression of immune-related genes in the cell nucleus. Such transcriptional activities triggered antimicrobial responses to eliminate the invading pathogens. The antimicrobial responses include activation of proPO system, the release of AMPs, and activation of phagocytosis.

At APm-T6, chitin deacetylase, galectin, HMGB1, STAT, Toll, IMD, serine proteinase, lysozyme, and apoptosis stimulating of p53 immune gene expressions were repressed. This is postulated to be the effects of DNA or cell damage inflicted by *Vp*_AHPND_ bacteria and released bacterial toxins. The transcriptional or pathway mechanisms were disrupted by the inflicted damage. Another possibility would be the hijacking and alteration of shrimp cell signalling or transcriptional mechanisms by *Vp*_AHPND_ bacteria. Despite that, this possibility was only mentioned briefly in past publication [23]. At the current moment, there is still an insufficient amount of studies on the probable hijacking of shrimp cell mechanism by bacteria as more focus is given to viral hijacking. Notably, at this time interval, techylectin was observed to be significantly upregulated. This suggests the occurrence of bacterial agglutination event to limit the pathogenicity and cytotoxicity of *Vp*_AHPND_ bacteria.

During APm-T24, due to the previously accumulated stress and cell damage condition, DAMP molecules were secreted to restore or further activate immune pathways and antimicrobial transcriptional activities. The sequential immunological responses described above continued on until the successful removal or elimination of *Vp*_AHPND_ bacteria from shrimp hepatopancreatic cells. In addition, the immunological response pattern may also be triggered by the invasion of *Vp*_AHPND_ bacteria at *P. monodon* intestinal cavities due to the important protein and hormone secretory role played by *P. monodon* hepatopancreas.

Other than that, the upregulation of antioxidant gene expressions at APm-T3 (Fig. 2) suggests the involvement of first line antioxidant defence. Glutathione-dependent prostaglandin d synthase and down syndrome cell adhesion molecule (DSCAM) gene expressions were upregulated indicating the activation of platelet or homologous mechanism and immune memory. Glutathione-dependent prostaglandin d synthase is important to prevent platelet aggregation [44] whereas DSCAM is a hypervariable protein importantly involved in shrimp innate immune memory. DSCAM displays a significantly elevated binding ability to the same pathogen associated with phagocytosis after repeated exposure. DSCAM’s immune priming to viruses has been previously validated [45], however, studies of DSCAM functioning in response to bacterial and fungal infections remain insufficient.

## Conclusion

As one of the commercial aquaculture shrimp species susceptible to AHPND infection and outbreak, the investigation of *P. monodon*’s innate immunity changes in response to AHPND infection is necessary for better understanding of the disease susceptibility. There have been previous transcriptomics studies of *P. monodon* challenged with *Vp*_AHPND_ bacteria, however, the focus is given to the major bacterial colonization site, which is the intestinal cavity [46]. Comparatively, hepatopancreas, which is an important colonization and toxin damage site for *Vp*_AHPND_, and hormone secretion site for shrimp is far less studied. In the present study, high-quality RNA-Seq transcriptome results were obtained from AHPND-challenged *P. monodon* hepatopancreas at different post-AHPND infection time intervals instead of intestine. The present study is also advantageous in terms of “time series treatment” transcriptomic analysis as compared to the common “untreated versus treated” transcriptomic analysis. The results successfully revealed the differentially expressed immune genes and inferred the overall sequential immunological response pattern of *P. monodon* hepatopancreas during AHPND infection.

In conclusion, there exists a systematic order of immune genes activation when in contact with bacterial PAMPs, involving pathogen-induced interactive response, PRRs, immune signalling pathways, AMPs, proPO system, phagocytosis, and other immune-related genes. The individual immune responses perform synergistically to achieve the maximum effect of pathogen elimination. By applying the fundamental knowledge obtained, specific genes can then be targeted for future applications of disease outbreak prevention, for example, detection of early pathogen infection, shrimp health diagnosis, and immune vaccination. Immune genes can be selected from different stages of the immune response to be developed as biomarkers and applied through different technologies, including PCR, qRT-PCR, loop-mediated isothermal amplification (LAMP), and enzyme-linked immunosorbent assay (ELISA). Recommended genes for biomarker applications are C-type lectin, IMD, ALF, HMGB1, serine proteinase, and DSCAM. Nevertheless, further validation works, such as protein expression analysis of important immune genes, can be done in the future to support the research findings.

## Additional file

**Additional file 1.** Transcriptomic analysis (RNA-Seq) details for AHPND-infected *P. monodon* hepatopancreas samples under APm-CTL, APm-T3, APm-T6, and APm-T24. **Table S1:** Summary of sequencing reads after filtering. **Table S2:** Transcriptome de novo assembly Unigenes quality metrics. **Figure S1:** Distribution of Unigenes based on FPKM values. **Figure S2:** Distribution of FPKM and TPM values based on length of Unigenes. **Table S3:** Overview of functional annotation results. **Figure S3:** Species distribution of transcriptome Unigenes annotated to NR annotation. **Figure S4:** Functional distribution of Unigenes based on COG analysis. **Figure S5:** Functional distribution of Unigenes based on GO annotation. **Figure S6:** Functional distribution of Unigenes based on KEGG annotation. **Figure S7:** Functionally enriched KEGG pathways based on DEGs. **Table S4.** Primer sequences designed for qRT-PCR analysis. **Figure S8:** Relative log2 fold change of *P. monodon* hepatopancreas C-type lectin gene expression across post-AHPND infection time points. **Table S5:** One-Way Analysis of Variance (One-Way ANOVA) and post hoc Duncan test showing statistical significance of *P. monodon* post-AHPND infection C-type Lectin relative gene expressions obtained. **Figure S9:** Relative log2 fold change of *P. monodon* hepatopancreas IMD gene expression across post-AHPND infection time points. **Table S6:** One-Way Analysis of Variance (One-Way ANOVA) and post hoc Duncan test showing statistical significance of *P. monodon* post-AHPND infection IMD relative gene expressions obtained. **Figure S10:** Relative log2 fold change of *P. monodon* hepatopancreas ALF lectin gene expression across post-AHPND infection time points. **Table S7:** One-Way Analysis of Variance (One-Way ANOVA) and post hoc Duncan test showing statistical significance of *P. monodon* post-AHPND infection ALF relative gene expressions obtained. **Figure S11:** Relative log2 fold change of *P. monodon* hepatopancreas HMGB1 lectin gene expression across post-AHPND infection time points. **Table S8:** One-Way Analysis of Variance (One-Way ANOVA) and post hoc Duncan test showing statistical significance of *P. monodon* post-AHPND infection HMGB1 relative gene expressions obtained.

## Data Availability

The datasets used and/or analysed during the current study are available from the corresponding author on reasonable request.

## Abbreviations

AHPND: acute hepatopancreatic necrosis disease
*Vp*_AHPND_: *Vibrio parahaemolyticus* AHPND strain
RNA-Seq: RNA sequencing
PCR: polymerase chain reaction
TSB+: tryptic soy broth supplemented with 2% sodium chloride (NaCl)
TSA+: tryptic soy agar supplemented with 2% sodium chloride (NaCl)
APm-CTL: AHPND-infected *P. monodon* control sample
APm-T3: 3 h post-AHPND infection *P. monodon* sample
APm-T6: 6 h post-AHPND infection *P. monodon* sample
APm-T24: 24 h post-AHPND infection *P. monodon* sample
FPKM: fragments per kilobase million
TPM: transcripts per million
NT: nucleotide database
NR: non-redundant protein sequence database
GO: Gene Ontology database
COG: Cluster of Orthologous Groups of proteins database
KEGG: Kyoto Encyclopedia of Genes and Genomes database
BLAST: Basic Local Alignment Search Tool
DEGs: differentially expressed genes
qRT-PCR: Real Time Reverse Transcription Polymerase Chain Reaction
One-Way ANOVA: One-Way Analysis of Variance
PAMPs: pathogen-associated molecular patterns
DAMPs: damage-associated molecular patterns
PRRs: pattern recognition receptor
AMPs: antimicrobial peptides
HMGB1: high mobility group box 1
JAK: Janus kinase
STAT: signal transducer and activator of transcription
IMD: immune deficiency
TLRs: toll-like receptors
TBK1: tank-binding kinase 1
STING: stimulator of interferon genes
ALRs: AIM2-like receptors
JNK: c-Jun N-terminal kinase
MAPK: P38 mitogen-activated protein kinase
IKK: IκB kinase
ALF: anti-lipopolysaccharide factor
proPO: prophenoloxidase
PO: phenoloxidase
SERPIN: serine proteinase inhibitor
DSCAM: down syndrome cell adhesion molecule
GlcNAc: β-1,4-linked *N*-acetylglucosamine
GbpA: GlcNAc-binding protein A
LPS: lipopolysaccharide
βG: β-1,3-glucan
PG: peptidoglycan
EF1a: elongation factor 1-alpha
LAMP: loop-mediated isothermal amplification
ELISA: enzyme-linked immunosorbent assay
