# Supplementary material for: Differential transcriptome analysis of the disease tolerant Madagascar–Malaysia crossbred black tiger shrimp, Penaeus monodon hepatopancreas in response to acute hepatopancreatic necrosis disease (AHPND) infection: inference on immune gene response and interaction
Source: Gut Pathog. 2019 Jul 26;11:39. doi: 10.1186/s13099-019-0319-4 (PMC6660963; doi:10.1186/s13099-019-0319-4)
Supplement: Supplementary file 1 — Additional file 1. Transcriptomic analysis (RNA-Seq) details for AHPND-infected P. monodon hepatopancreas samples under APm-CTL, APm-T3, APm-T6, and APm-T24. Table S1: Summary of sequencing reads after filtering. Table S2: Transcriptome de novo assembly Unigenes quality metrics. Figure S1: Distribution of Unigenes based on FPKM values. Figure S2: Distribution of FPKM and TPM values based on length of Unigenes. Table S3: Overview of functional annotation results. Figure S3: Species distribution of transcriptome Unigenes annotated to NR annotation. Figure S4: Functional distribution of Unigenes based on COG analysis. Figure S5: Functional distribution of Unigenes based on GO annotation. Figure S6: Functional distribution of Unigenes based on KEGG annotation. Figure S7: Functionally enriched KEGG pathways based on DEGs. Table S4. Primer sequences designed for qRT-PCR analysis. Figure S8: Relative log2 fold change of P. monodon hepatopancreas C-type lectin gene expression across post-AHPND infection time points. Table S5: One-Way Analysis of Variance (One-Way ANOVA) and post hoc Duncan test showing statistical significance of P. monodon post-AHPND infection C-type Lectin relative gene expressions obtained. Figure S9: Relative log2 fold change of P. monodon hepatopancreas IMD gene expression across post-AHPND infection time points. Table S6: One-Way Analysis of Variance (One-Way ANOVA) and post hoc Duncan test showing statistical significance of P. monodon post-AHPND infection IMD relative gene expressions obtained. Figure S10: Relative log2 fold change of P. monodon hepatopancreas ALF lectin gene expression across post-AHPND infection time points. Table S7: One-Way Analysis of Variance (One-Way ANOVA) and post hoc Duncan test showing statistical significance of P. monodon post-AHPND infection ALF relative gene expressions obtained. Figure S11: Relative log2 fold change of P. monodon hepatopancreas HMGB1 lectin gene expression across post-AHPND infection time points. Tab [file 13099_2019_319_MOESM1_ESM.docx]

**Table S1: Summary of sequencing reads after filtering.**

| **Sample** | **Total Raw Reads (Mb)** | **Total Clean Reads (Mb)** | **Total Clean Bases (Gb)** | **Clean Reads Q30 (%)** | **Clean Reads Ratio (%)** |
| --- | --- | --- | --- | --- | --- |
| APm-CTL | 80.16 | 78.2 | 7.82 | 91.46 | 97.56 |
| APm-T3 | 80.29 | 78.13 | 7.81 | 91.31 | 97.31 |
| APm-T6 | 80.02 | 78.29 | 7.83 | 92.18 | 97.84 |
| APm-T24 | 80.1 | 78.15 | 7.81 | 91.35 | 97.57 |
| Total | 320.57 | 312.77 | 31.27 | - | - |

**Table S2: Transcriptome *de novo* assembly Unigenes quality metrics.**

| **Sample** | **Total Number** | **Total Length** | **Mean Length** | **N50** | **N70** | **N90** | **GC (%)** |
| --- | --- | --- | --- | --- | --- | --- | --- |
| APm-CTL | 33222 | 30504667 | 918 | 1714 | 880 | 337 | 44.16 |
| APm-T3 | 41527 | 41092534 | 989 | 1902 | 1015 | 357 | 44.03 |
| APm-T6 | 36865 | 34636710 | 939 | 1794 | 928 | 338 | 44.10 |
| APm-T24 | 42649 | 42772885 | 1002 | 1939 | 1033 | 359 | 43.89 |
| All-Unigene | 48662 | 56085297 | 1152 | 2289 | 1294 | 412 | 44.10 |


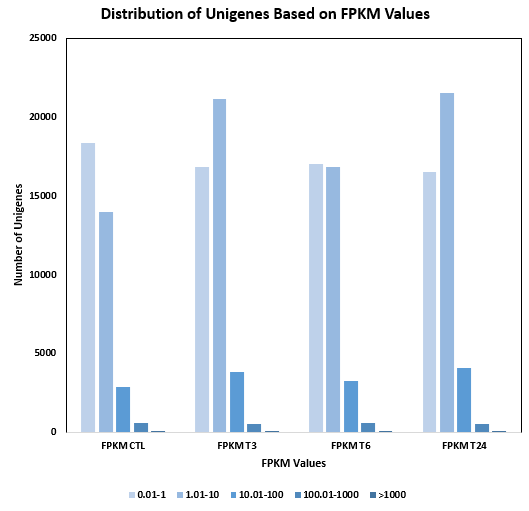


**Figure S1: Distribution of Unigenes based on FPKM values.**


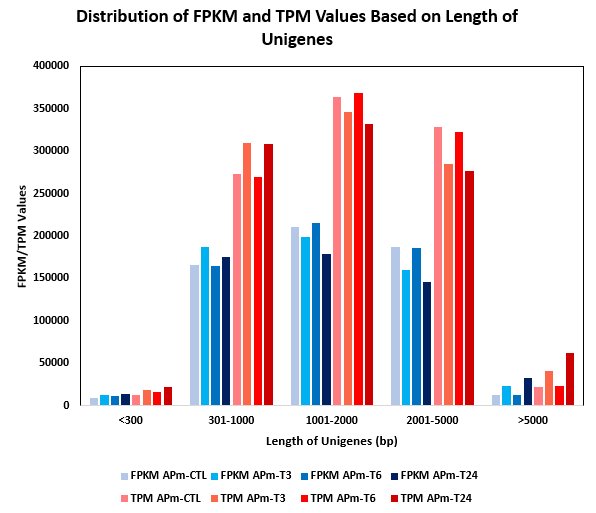


**Figure S2: Distribution of FPKM and TPM values based on Length of Unigenes.**

**Table S3: Overview of functional annotation results.**

| **Values** | **Number** | **Percentage** |
| --- | --- | --- |
| Unigenes Annotated to Nr | 24,709 | 50.78% |
| Unigenes Annotated to Nt | 18,622 | 38.27% |
| Unigenes Annotated to SwissProt | 21,388 | 43.95% |
| Unigenes Annotated to COG | 11,636 | 23.91% |
| Unigenes Annotated to KEGG | 20,696 | 42.53% |
| Unigenes Annotated to GO | 4,510 | 9.27% |
| Unigenes Annotated to InterPro | 16,438 | 33.78% |
| Overall | 29,106 | 59.81% |
| Total | 48,662 | 100% |


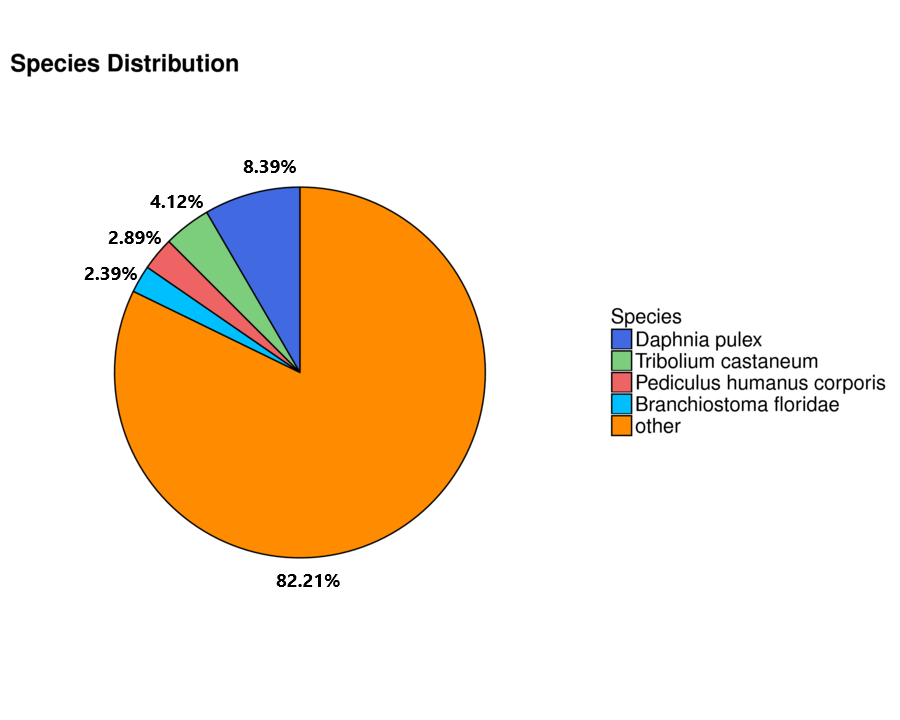


**Figure S3: Species distribution of transcriptome Unigenes annotated to NR annotation.**


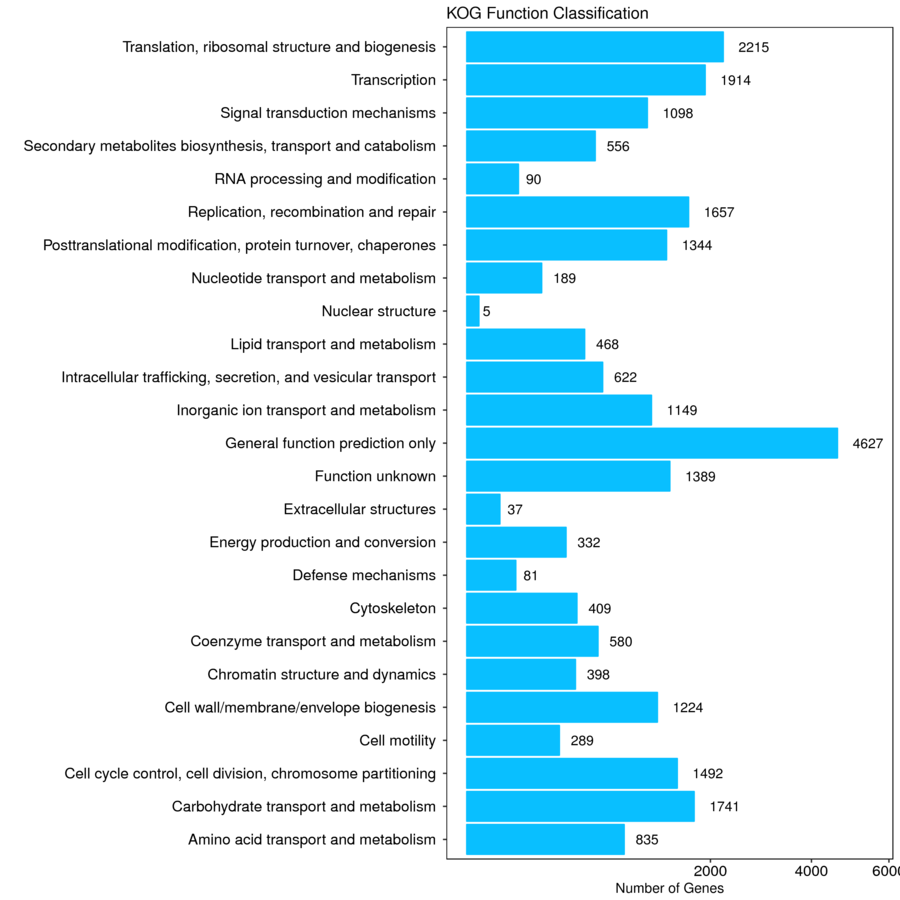


**Figure S4: Functional distribution of Unigenes based on COG analysis.**


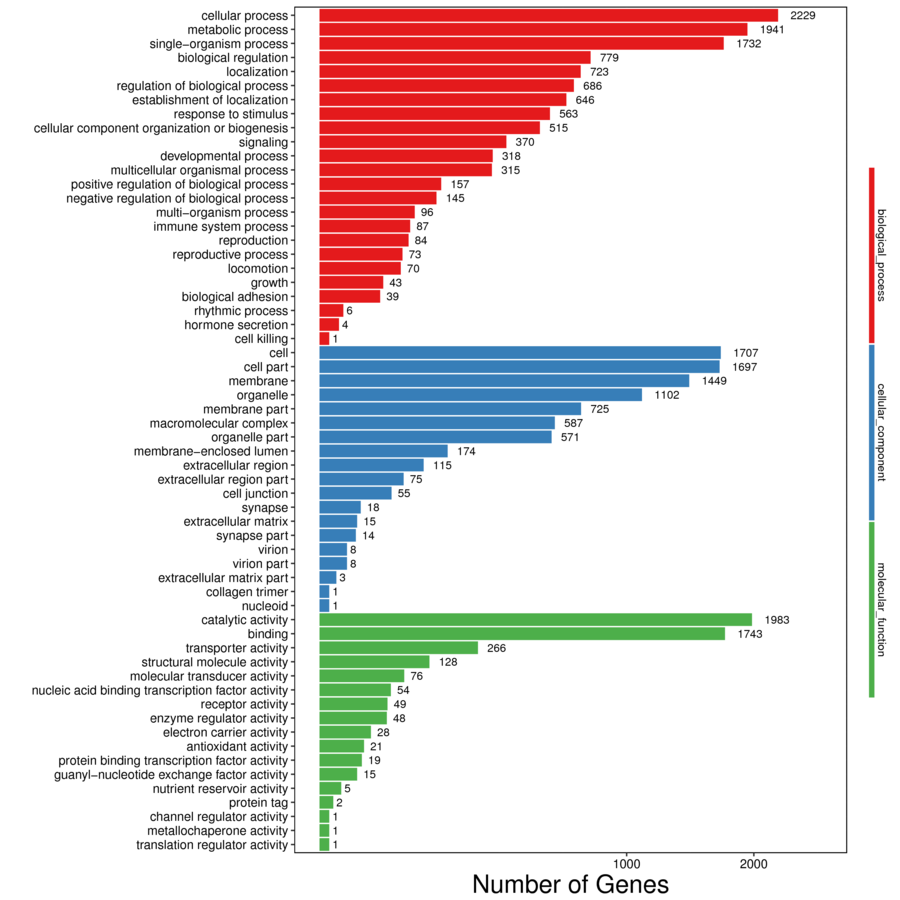


**Figure S5: Functional distribution of Unigenes based on GO annotation.**


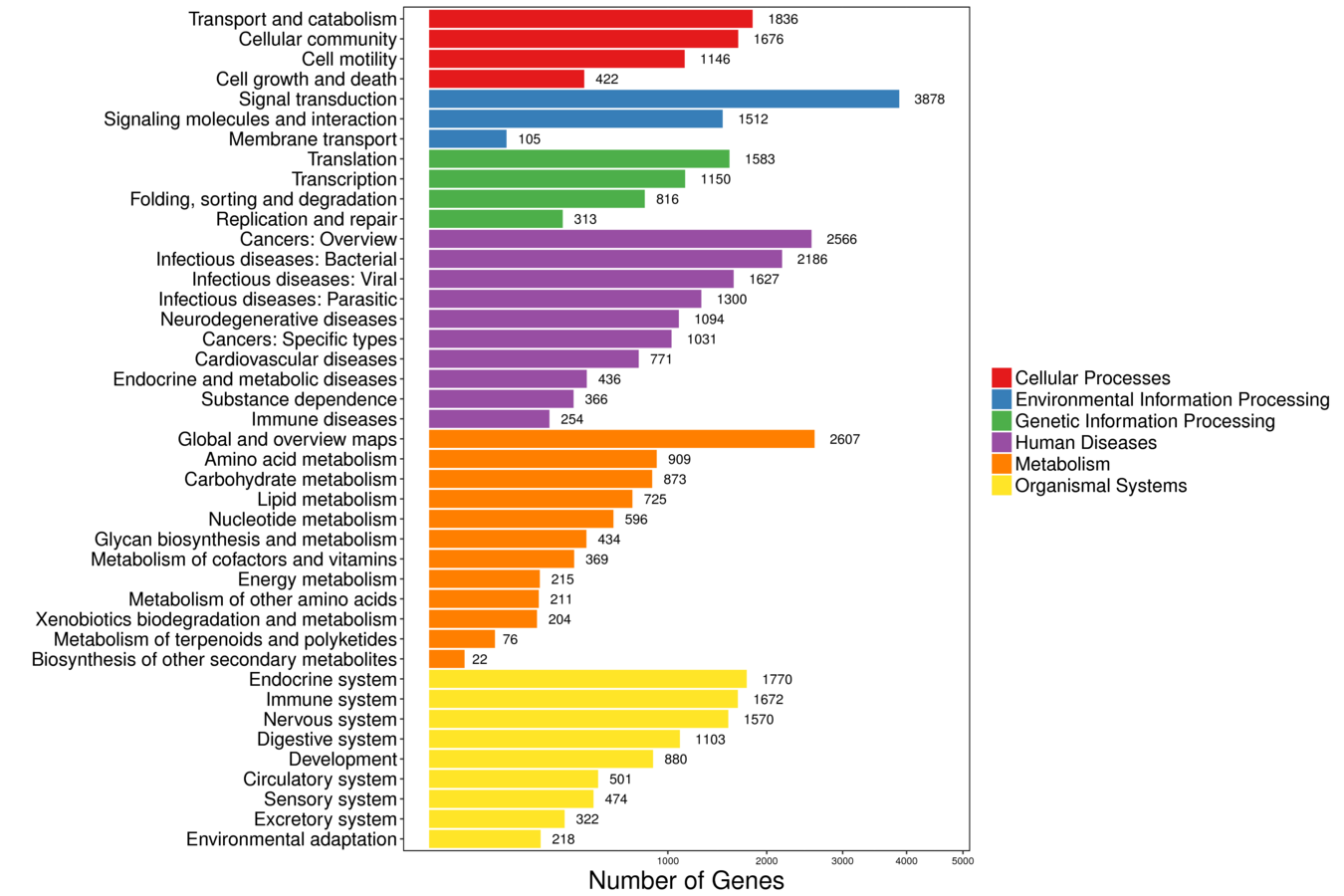


**Figure S6: Functional distribution of Unigenes based on KEGG annotation.**


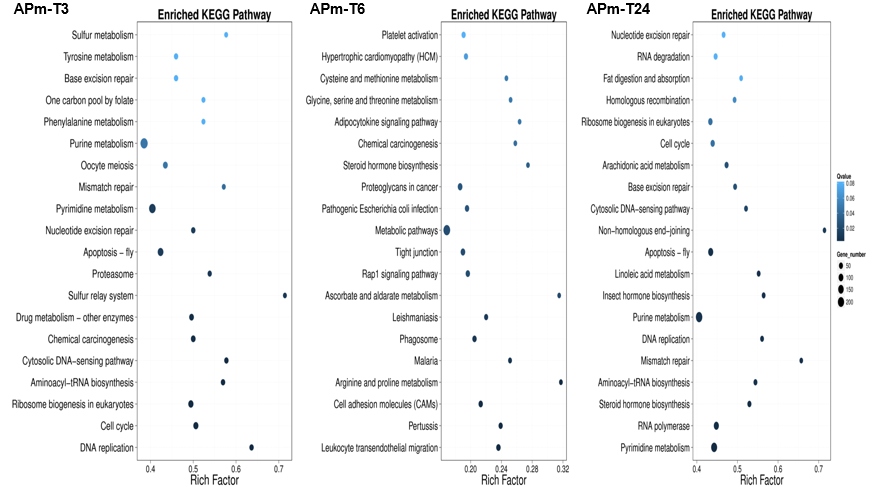


**Figure S7: Functionally enriched KEGG pathways based on DEGs.**

**Table S4: Primer sequences designed for qRT-PCR analysis.**

| **No.** | **Primer Sequence** | **Target Gene** |
| --- | --- | --- |
| 1 | **qPmCTL-F:** 5’- GAA CGT GGA ACT GGC TGG AC -3’ | C-type lectin |
| 2 | **qPmCTL-R:** 5’- CTG GTA ATC GTT GAA GGT GGG ATG -3’ | C-type lectin |
| 3 | **qPmIMD-F:** 5’- CGA GAC AAG GTC GAG GTC AG -3’ | IMD |
| 4 | **qPmIMD-R:** 5’- CTC GTA CAC TCG GTC GAC ATT A -3’ | IMD |
| 5 | **qPmALF-F:** 5’- GCC ACC ACA AGA ACC TTA GA -3’ | ALF |
| 6 | **qPmALF-R:** 5’- ACT GCT CCG GTT TAG AGA AAG -3’ | ALF |
| 7 | **qPmHG-F:** 5’- TTC ATC CCG ACG AGA ATG TG -3’ | HMGB1 |
| 8 | **qPmHG-R:** 5’- CCA TGT CGT GGA ACT TCT TCT -3’ | HMGB1 |
| 9 | **qELF F**: 5’- TATGGTTGTCAACTTTGCCCC -3’ | EF1a |
| 10 | **qELF R**: 5’- AACCTCGCTTCAGATCCTTTAC -3’ | EF1a |


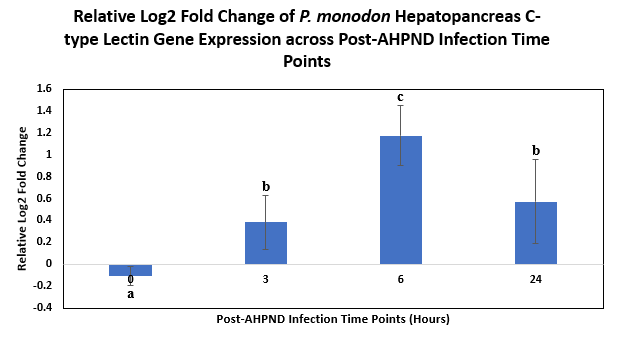


**Figure S8: Relative log2 fold change of *P. monodon* hepatopancreas C-type lectin gene expression across post-AHPND infection time points.**

**Table S5: One-Way Analysis of Variance (One-Way ANOVA) and post hoc Duncan test showing statistical significance of *P. monodon* post-AHPND infection C-type Lectin relative gene expressions obtained.**

| **ANOVA** | | | | | |
| --- | --- | --- | --- | --- | --- |
| Relative Gene Expression | | | | | |
|  | Sum of Squares | df | Mean Square | F | Sig. |
| Between Groups | 2.520 | 3 | .840 | 11.528 | .003 |
| Within Groups | .583 | 8 | .073 |  |  |
| Total | 3.102 | 11 |  |  |  |

| **Relative Gene Expression** | | | | |
| --- | --- | --- | --- | --- |
| Duncan^a^ | | | | |
| Time Hours Post-AHPND Infection | N | Subset for alpha = 0.05 | | |
|  |  | a | b | c |
| 0 | 3 | -.10667 |  |  |
| 3 | 3 | .38333 | .38333 |  |
| 24 | 3 |  | .57000 |  |
| 6 | 3 |  |  | 1.17333 |
| Sig. |  | .057 | .422 | 1.000 |
| Means for groups in homogeneous subsets are displayed. | | | | |
| a. Uses Harmonic Mean Sample Size = 3.000. | | | | |


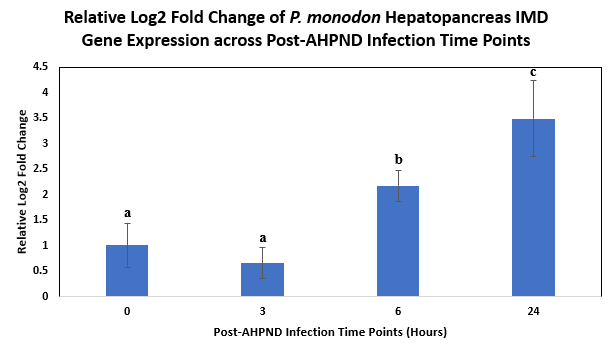


**Figure S9: Relative log2 fold change of *P. monodon* hepatopancreas IMD gene expression across post-AHPND infection time points.**

**Table S6: One-Way Analysis of Variance (One-Way ANOVA) and post hoc Duncan test showing statistical significance of *P. monodon* post-AHPND infection IMD relative gene expressions obtained.**

| **ANOVA** | | | | | |
| --- | --- | --- | --- | --- | --- |
| Relative Gene Expression | | | | | |
|  | Sum of Squares | df | Mean Square | F | Sig. |
| Between Groups | 14.794 | 3 | 4.931 | 21.158 | <0.001 |
| Within Groups | 1.865 | 8 | .233 |  |  |
| Total | 16.658 | 11 |  |  |  |

| **Relative Gene Expression** | | | | |
| --- | --- | --- | --- | --- |
| Duncan^a^ | | | | |
| Time Hours Post-AHPND Infection | N | Subset for alpha = 0.05 | | |
|  |  | a | b | c |
| 3 | 3 | .66000 |  |  |
| 0 | 3 | 1.00667 |  |  |
| 6 | 3 |  | 2.17333 |  |
| 24 | 3 |  |  | 3.49333 |
| Sig. |  | .405 | 1.000 | 1.000 |
| Means for groups in homogeneous subsets are displayed. | | | | |
| a. Uses Harmonic Mean Sample Size = 3.000. | | | | |


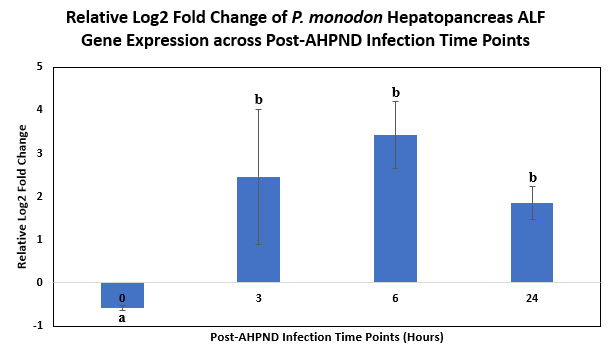


**Figure S10: Relative log2 fold change of *P. monodon* hepatopancreas ALF lectin gene expression across post-AHPND infection time points.**

**Table S7: One-Way Analysis of Variance (One-Way ANOVA) and post hoc Duncan test showing statistical significance of *P. monodon* post-AHPND infection ALF relative gene expressions obtained.**

| **ANOVA** | | | | | |
| --- | --- | --- | --- | --- | --- |
| Relative Gene Expression | | | | | |
|  | Sum of Squares | df | Mean Square | F | Sig. |
| Between Groups | 26.198 | 3 | 8.733 | 10.883 | .003 |
| Within Groups | 6.419 | 8 | .802 |  |  |
| Total | 32.617 | 11 |  |  |  |

| **Relative Gene Expression** | | | |
| --- | --- | --- | --- |
| Duncan^a^ | | | |
| Time Hours Post-AHPND Infection | N | Subset for alpha = 0.05 | |
|  |  | a | b |
| 0 | 3 | -.58000 |  |
| 24 | 3 |  | 1.85000 |
| 3 | 3 |  | 2.45667 |
| 6 | 3 |  | 3.42333 |
| Sig. |  | 1.000 | .073 |
| Means for groups in homogeneous subsets are displayed. | | | |
| a. Uses Harmonic Mean Sample Size = 3.000. | | | |


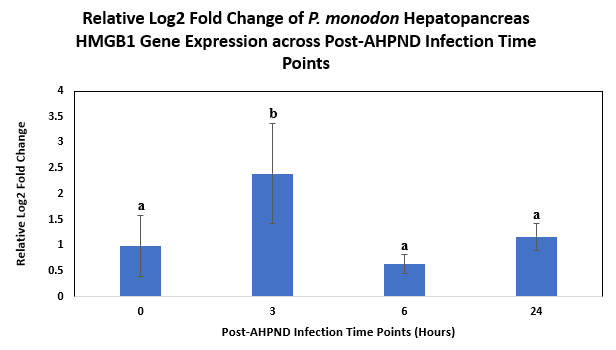


**Figure S11: Relative log2 fold change of *P. monodon* hepatopancreas HMGB1 lectin gene expression across post-AHPND infection time points.**

**Table S8: One-Way Analysis of Variance (One-Way ANOVA) and post hoc Duncan test showing statistical significance of *P. monodon* post-AHPND infection HMGB1 relative gene expressions obtained.**

| **ANOVA** | | | | | |
| --- | --- | --- | --- | --- | --- |
| Relative Gene Expression | | | | | |
|  | Sum of Squares | df | Mean Square | F | Sig. |
| Between Groups | 5.254 | 3 | 1.751 | 5.024 | .030 |
| Within Groups | 2.789 | 8 | .349 |  |  |
| Total | 8.042 | 11 |  |  |  |

| **Relative Gene Expression** | | | |
| --- | --- | --- | --- |
| Duncan^a^ | | | |
| Time Hours Post-AHPND Infection | N | Subset for alpha = 0.05 | |
|  |  | a | b |
| 6 | 3 | .64000 |  |
| 0 | 3 | .99000 |  |
| 24 | 3 | 1.17000 |  |
| 3 | 3 |  | 2.39667 |
| Sig. |  | .322 | 1.000 |
| Means for groups in homogeneous subsets are displayed. | | | |
| a. Uses Harmonic Mean Sample Size = 3.000. | | | |
